# Potential Mechanisms of Precision Nutrition-Based Interventions for Managing Obesity

**DOI:** 10.1016/j.advnut.2024.100186

**Published:** 2024-02-03

**Authors:** Neel H Mehta, Samantha L Huey, Rebecca Kuriyan, Juan Pablo Peña-Rosas, Julia L Finkelstein, Sangeeta Kashyap, Saurabh Mehta

**Affiliations:** 1 Division of Nutritional Sciences, Cornell University, Ithaca, NY, United States; 2 Center for Precision Nutrition and Health, Cornell University, Ithaca, NY, United States; 3 Division of Nutrition, St. John’s Research Institute, Bengaluru, Karnataka, India; 4 Global Initiatives, The Department of Nutrition and Food Safety, World Health Organization, Geneva, Switzerland; 5 Division of Endocrinology, Diabetes and Metabolism, Weill Cornell Medicine New York Presbyterian, New York, NY, United States; 6 Division of Medical Informatics, St. John’s Research Institute, Bengaluru, Karnataka, India

**Keywords:** personalized nutrition, overweight, adiposity, prevention, interventions, randomized trials

## Abstract

Precision nutrition (PN) considers multiple individual-level and environmental characteristics or variables to better inform dietary strategies and interventions for optimizing health, including managing obesity and metabolic disorders. Here, we review the evidence on potential mechanisms—including ones to identify individuals most likely to respond—that can be leveraged in the development of PN interventions addressing obesity. We conducted a review of the literature and included laboratory, animal, and human studies evaluating biochemical and genetic data, completed and ongoing clinical trials, and public programs in this review. Our analysis describes the potential mechanisms related to 6 domains including genetic predisposition, circadian rhythms, physical activity and sedentary behavior, metabolomics, the gut microbiome, and behavioral and socioeconomic characteristics, i.e., the factors that can be leveraged to design PN-based interventions to prevent and treat obesity-related outcomes such as weight loss or metabolic health as laid out by the NIH 2030 Strategic Plan for Nutrition Research. For example, single nucleotide polymorphisms can modify responses to certain dietary interventions, and epigenetic modulation of obesity risk via physical activity patterns and macronutrient intake have also been demonstrated. Additionally, we identified limitations including questions of equitable implementation across a limited number of clinical trials. These include the limited ability of current PN interventions to address systemic influences such as supply chains and food distribution, healthcare systems, racial or cultural inequities, and economic disparities, particularly when designing and implementing PN interventions in low- and middle-income communities. PN has the potential to help manage obesity by addressing intra- and inter-individual variation as well as context, as opposed to “one-size fits all” approaches though there is limited clinical trial evidence to date.

**Statements of Significance**

Optimizing dietary strategies and interventions at the individual level through genetic and microbiome assessment, lifestyle pattern analysis, and phenotyping may help advance our ability to modulate and/or manage individual physiological factors involved in obesity.

## Introduction

Obesity is among today’s most pressing global health challenges, with enormous human and economic costs. The WHO estimated that in 2016, over 650 million adults had obesity and an additional 340 million adolescents had either overweight or obesity, and in 2020, nearly 40 million children had overweight or obesity [1]. The high prevalence of obesity results in a financial and socioeconomic burden on the healthcare system; for example, ∼$260.6 billion USD in costs is placed on the United States healthcare system due to disease burden and downstream health effects [2]. Among adults, high BMI is a major risk factor for noncommunicable diseases including cardiovascular diseases, metabolic diseases such as diabetes, musculoskeletal disorders, and certain cancers such as those related to the reproductive and gastrointestinal systems [3]. In children, obesity is associated with respiratory issues such as asthma and sleep apnea, neurological disorders including intracranial hypertension, and decreased psychosocial health related to weight stigmas and self-esteem [4]. Long-term consequences of pediatric obesity include increased risk of multiple degenerative and autoimmune disorders, including type 2 diabetes, multiple sclerosis, Crohn’s disease, and arthritis [5]. Current efforts to manage obesity in adults range from surgical interventions including bariatric surgeries such as gastric bypass or sleeve gastrectomy [6], which have stringent eligibility criteria such as a BMI >40 or between 35 and 40 kg/m^2^ with associated comorbidities [7]; pharmacotherapies such as orlistat, metformin, phentermine, glucagon-like peptide-1 agonists, and naltrexone [[8], [9], [10], [11]]; and behavior-change interventions such as counseling to adjust diet [12], along with lifestyle modifications. However, surgical and medicinal interventions are expensive and not always covered by insurance, and may come with complications or side effects, limiting the accessibility of such interventions to only a minority of patients [13].

**FIGURE 1:**
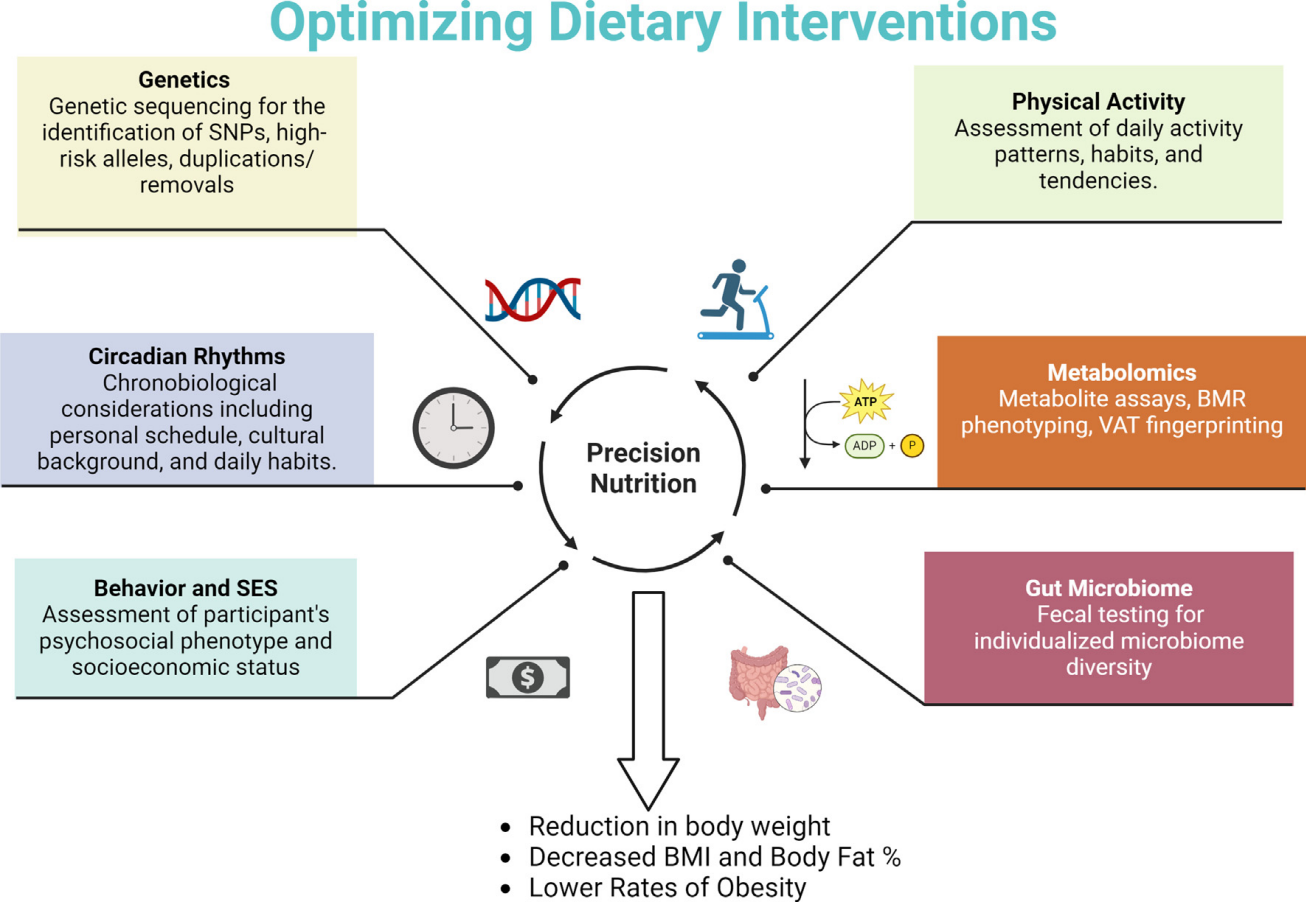
Schematic diagram of optimization of precision nutrition intervention. Precision nutrition interventions can be optimized through integration of genetics, circadian rhythms, behavior and socioeconomic status, physical activity patterns, metabolomics, and gut microbiota diversity. Optimized precision interventions are proposed to reduce rates of obesity and improve measures of body composition. BMR, basal metabolic rate; SES, socioeconomic status; SNP, single nucleotide polymorphism; VAT, visceral adipose tissue.

Clinical trials examining “one-size-fits-all” diets, which we define as a general dietary pattern applied to all participants in a group, such as one high-fat, low-carbohydrate, high-protein, etc., low-calorie diet [14], have observed interindividual variation in blood glucose levels in response to eating the same food [15,16]. Glucose response is a significant risk factor for diabetes and is also linked to obesity and metabolic syndrome [17]; if a given food results in a low glucose response for individual A but a high response in individual B, then it may be useful to only recommend that food to individual A, not B. Individual-level glucose responses were predicted with high accuracy by using a machine learning algorithm to incorporate multiple individual-level clinical, biological, and physical characteristics [18,19]. Further, participants fared better on a diet personalized for them using this algorithm compared to a standard diet designed to lower the glucose response without accounting for individual-level characteristics [19]. Tailoring a diet specific to each individual is known as precision or personalized nutrition (PN) [20]. Although there is some debate about the difference between the 2 terms, popular usage does not tend to differentiate between precision and personalized nutrition. Specifically, PN is a comprehensive consideration of individual-level and environmental characteristics or domains such as genetic predisposition, circadian rhythms, physical activity and sedentary behavior, metabolomics, the gut microbiome, and behavioral and socioeconomic characteristics, some of which are included in the Strategic Plan (2020–2030) for NIH Nutrition Research [21]. These domains can be assessed using a combination of clinical, bioinformatic, and genetic approaches, such as nutrigenomics, microbiome, and deep phenotyping [22], and can be used to develop personalized interventions or diet plans catered to individual nutritional needs to address metabolic syndrome and obesity [22]. Importantly, these domains are not exhaustive; may be influenced by other considerations not explicitly discussed here, such as climate change, food systems, and the built environment; and reflect considerable variability in the consistency and quality of evidence. As such, the objective of this review is to provide a summary of the current state of PN, examining how variables within each of these domains are mechanistically tied to the outcome of interest, obesity management, and contribute to ultimately the design of PN approaches for decreasing obesity prevalence and severity (Figure 1). Our bird’s eye view of PN discusses the interrelatedness of variables within and between each domain, adverse effects, limitations, and challenges to PN as an intervention for obesity, while highlighting the future directions and considerations for the field.

## Genetics

Although genetic variation only explains a small proportion of risk of obesity in the population, it may confer a tendency for weight gain and development of adiposity-based chronic diseases based on exposure to diet and external factors [23]. Considering the biochemical basis of obesity, the impact of individual genetics—from single nucleotide polymorphisms (SNPs) to allelic frequencies—on the predisposition to and perpetuation of obesity may provide a biological framework in the design of PN interventions. In animal models, genome-wide association studies (GWASs) have identified quantitative trait loci (QTLs) that map with confidence to various phenotypic outcomes. In one such study, in silico QTL mapping was used to identify 937 QTLs across 173 mouse phenotypes, with the *Adam12* and *Cdh2* genes (associated with percent of fat mass and liver weight, respectively, after 8 wk on an atherogenic diet) being associated with diet-linked obesity [24]. High-resolution QTL mapping resolved tightly linked loci to achieve single gene resolution, which may be utilized to better understand the polygenic risk of obesity. Similar GWASs in mouse models of obesity have identified obesity-related genes on the proximal chromosome 13 [[25], [26], [27]].

Conducting GWASs in animal models has allowed for extensive genome-wide association analyses in humans that reveal the involvement of multiple loci and gene products as potential mechanisms in obesity [28]. In one study, a sample of nearly 340,000 subjects was included in a GWAS that identified 97 loci associated with increased BMI [29]. These loci accounted for 2.7% of BMI variation and provided evidence that 21% of variation in BMI can be attributed to natural genetic variation [29]. On the mechanistic level, the study emphasized how gene products, such as brain-derived neurotrophic factor and melanocortin 4 receptor, function in hunger homeostasis via regulation of glutamate receptor activity in the hypothalamus [29]. These genetic variations of key metabolites and proteins may represent an important node of optimization for PN-based dietary interventions that can be examined at the individual level via gene and gene product assays.

The molecular and genetic predispositions to obesity in humans have also been understood directly in the context of feeding behaviors, dietary selection, and nutrient intake. In one study, a genetic predisposition score was calculated using 32 SNPs known to be associated with obesity, which was used to examine the impact of increased intake of sugar-sweetened beverages [30]. Here, the genetic burden on BMI and obesity was nearly doubled in participants consuming >1 sugary beverage daily compared to subjects consuming <1 sugary beverage on average per month [30]. In other words, loci implicated in the development of obesity appeared epigenetically regulated by the nutritional composition of sugary beverages [30], marking another example in which individualized polygenic risk may be useful in the development of novel and personalized nutrition plans. A similar GWAS used 63 obesity-related loci to create and compare a polygenic risk score with the intake of saturated fatty acids [31]. Results indicated that key genetic loci, including the *FTO* and *APOA2* genes, both hypothalamic regulators of hunger, had increased allelic variation in individuals consuming more saturated fatty acids [31]. In this case, dietary modulation of certain genetic loci may have resulted in greater susceptibility to obesity. Further, being active was shown to lessen the impact of allelic variation within the *FTO* gene (variant rs1121980) that increases risk for obesity development: sedentary or inactive individuals with risk (T) allele had greater BMI indices than those engaging in physical activity [22,32]. The regulation of the genome and subsequent neuroendocrine control of obesity appears to be associated with the nutritional composition and frequency of an individual’s diet as well as physical activity level [22,[32], [33], [34], [35]].

Furthermore, as summarized by Heianza et al. [28], individual genetic variation can change the effect of high-protein diets and low-fat/high-carbohydrate diets in subjects with obesity and overweight status. Individuals with overweight without the C allele of *PPM1K* rs1440581 SNP responded better to a high-fat diet, due to dysregulation of branched-chain and aromatic amino acids [36]. Similarly, individuals with the CC genotype of *RS1* rs2943641 SNP were better suited to a high-carbohydrate/low-fat diet than control counterparts [37]. These genetically predisposed and disparate responses to dietary interventions may be rooted in a reciprocal relationship between macronutrient intake and genetic SNPs in which epigenetic modifications from nutrient intake and genetic regulation of nutrient absorption pathways occur simultaneously. For example, one study used genetic risk scores (GRSs) from 16 SNPs related to obesity or lipid metabolism to demonstrate that high genetic risk correlated with increased adiposity, and macronutrient intake (proteins, total fat, saturated fatty acids, polyunsaturated fats, total and complex carbohydrates, and fiber) can epigenetically modify these associations [38]. A similar study of 13 replicated SNPs related to GRS with macronutrient intake, associated increased GRS with higher fiber intake and lower total energy intake. The study, however, did not identify relationships between macronutrient intake levels and modified genetic predisposition to obesity, emphasizing the need for further research that better contextualizes genetic risk, epigenetic regulation, and dietary modifications [39]. There is a need to comprehensively understand the molecular and genetic predispositions to obesity and how these are epigenetically modified, which may directly inform an individual’s response to dietary interventions. Prospective randomized analyses of GWASs and alternative genome sequencing studies may provide insights into the effective and practical development of PN plans. Additional studies in children and adolescents are required to clarify the importance, or lack thereof, of these genomic associations across the human lifespan.

## Circadian Rhythms

Although genetic information encodes the molecular and biochemical pathways that can predispose an individual to obesity, the regulation and homeostasis of various hormones, signaling factors, and cells provides a temporal modulation of healthy metabolism and obesity pathogenesis [40]. To understand the physiology of obesity, the role of circadian rhythms—natural fluctuations in signaling pathways and molecules—must be appreciated [41]. Circadian rhythms, defined as an individual’s “internal clock,” is largely set by the suprachiasmatic nucleus (SCN) of the hypothalamus and is partially involved in parasympathetic-sympathetic regulation, metabolite cycling, and endocrine activation [41]. In humans, this machinery is set into motion when light transduced in photoreceptors of the retina activates thousands of neurons in the SCN, causing the dimerization of 2 fundamental transcription factors: BMAL1 and CLOCK [42]. Activation of the BMAL1-CLOCK complex induces transcription of negative regulators such as period (PER) and cryptochrome, whose subsequent production serves to downregulate transcriptions of various downstream genes [42]. Activation of the PER and cryptochrome transcriptional regulation also deactivates the BMAL1-CLOCK complex, resulting in a negative feedback loop susceptible to diurnal variations in the circadian rhythm [42]. In this manner, the circadian machinery creates diurnal feedback loops to activate and deactivate genomic transcription, and in doing so, serves to regulate downstream metabolic and physiologic activity. The impact of circadian rhythms on physiological wellness is largely studied with chronobiological techniques aimed at understanding the biochemical mechanisms of temporal regulation in nondiseased biology and disease pathways [43].

At the molecular level, an array of literature has examined the circadian regulation of metabolite activity, body composition, and predisposition to metabolic disorders. One such study analyzed over 500 liver metabolites in mice using ultrahigh performance liquid chromatography with tandem mass spectrometry to identify the diurnal variations in metabolite concentrations [44]. Findings indicated a strong temporal bias of metabolite levels, suggesting a highly regulated downstream metabolic activity [44]. Similar transcriptomic and proteomic analyses in circadian mutant mice displayed distinct temporal effects on liver metabolite concentrations and genetic transcription rate [45,46]. This epigenetic regulation of metabolite physiology has been reflected in direct associations between circadian rhythm dysfunction and a variety of disease states including obesity [47], type 2 diabetes [48], and metabolic syndrome [49].

Although the circadian clock machinery has been studied as a facilitator for disease pathogenesis, the reciprocal regulation of circadian rhythms via nutritional intake and dietary choices has also been examined. In one example of this metabolic regulation of cellular time, in vitro glucose enrichment of rat fibroblasts was found to downregulate PER1 and PER2 expression levels. Here, 2 variants of these transcription factors were responsible for downstream mediation of a peripheral cellular clock [50]. Similarly, another study in rats found glucose actively suppressed the natural circadian activation of the enzyme phosphoenolpyruvate carboxykinase, a key enzyme in gluconeogenesis, suggesting that high-sugar diets may also fundamentally alter the natural metabolic rhythms of the body [51]. Another investigation of the impact of diet on SCN-mediated circadian rhythms found temporally restricted light-dark or dark-dark feeding restrictions to uncouple central nervous circadian control from a variety of peripheral cell types and encourage dysregulation of hunger [52]. As such, the disruption of these homeostatic physiological systems, grounded in bidirectional crosstalk between dietary needs and genetic transcription, play a role in metabolic control, hunger regulation, and gastrointestinal disease [53].

These preclinical and in vitro studies have set the stage for the further study of the relationships between circadian biology, dietary habits, and human health. Recent epidemiological and experimental studies have highlighted how food choices that lead to diets high in fat and carbohydrates can lead to impaired circadian clocks with sleep impairment [54,55]. Additionally, social pressures and habits that encourage chronic sleep deprivation have been shown to drive calorie-dense food choices that increase energy and food intake, resulting in a higher risk and prevalence of obesity [56]. Social pressures toward dysregulated circadian rhythms are numerous and may range from late night shift work [57] to chronic stress-mediated lack of sleep, emphasizing the need to integrate these social and behavioral influences with circadian considerations for PN. Importantly, functional MRI studies have suggested that sleep deprivation can modulate olfactory neural circuits to induce a preference for calorie-dense foods, supporting the potential role of irregular mealtimes and sleep schedules as contributors to developing overweight and obesity [58]. On the mechanistic level, circadian biology is governed by glucose homeostasis via hormonal signaling, and alterations in sleep cycles, dietary habits, and molecular variations can impact risk of developing metabolic disease. In this sense, understanding the timing of food intake and its bidirectional relationship with sleep cycles and circadian rhythms may be an important consideration in improving the design of personalized interventions to improve clinical outcomes [59]. With our growing understanding of gene-diet interactions, circadian dysregulation, and disease pathogenesis, a focus on the temporal optimization of dietary interventions may serve as the target question of future prospective studies [53]. As such, appreciating the bidirectional relationships between dietary habits and circadian rhythms will be key to developing the necessary biomarkers, tools, and assessments to optimize personalized interventions [59]. Additional large-scale randomized trials in adults and children may examine the effects of these natural temporal variations in driving the adoption, acceptability, and physiological response to PN interventions. Appreciating an individual’s circadian rhythm may provide novel insights into the biophysiological systems that mediate obesity, and tailoring dietary interventions to both account for and work with these natural dietary rhythms may facilitate an increase in intervention efficacy. These considerations of sleep patterns, circadian rhythms, dietary habits, and energy balance can help identify the effects of adapting the timing and composition of dietary interventions based on healthy eating and sleep patterns on an individualized basis and may improve the efficacy of and adherence to these key PN-based interventions.

## Physical Activity and Sedentary Behavior

Sedentary behavior and physical activity remain 2 of the leading modifiable risk factors for metabolic and cardiovascular disease. Greater levels and patterns of physical activity lead to improved cardiovascular function, better nutritional digestion and intake, and optimal caloric utilization, all of which contribute to diminished rates of obesity [60]. Optimal PN dietary interventions must account for physical activity patterns and cultural habits that often mediate the sociocultural and physiological development of obesity. Large-scale ecological, cross-sectional, and prospective studies have reported an almost unanimous concern with the effects of a sedentary lifestyle in promoting weight gain and subsequent obesity [61]. Recent efforts in the field of “sedentary physiology” suggest that a sedentary lifestyle may induce activation of the sympathetic nervous system, influencing enzymatic activity, hormonal regulation of hunger and digestion, and general cardiovascular health [62]. At the molecular level, transcriptomic analyses have connected chronic inactivity with epigenetic regulation of microRNA (miRNA)-222, miRNA-146, miRNA-16, miRNA-126, and miR-320, all molecular contributors to immune pathways or cardiovascular health [63]. Further, there has also been documentation of adverse responses, consisting of exercise-induced worsening of fasting plasma HDL-cholesterol, triglycerides, blood pressure, or insulin, following PN interventions, suggesting that study of individualized physical activity profiles is needed to identify predictors of intervention response [64]. As such, personalized attempts to understand the intensity and frequency of physical activity, or lack thereof, are fundamental in capturing the complete genetic, epigenetic, and sociological causes of obesity.

A 24-wk interventional study of 171 sedentary middle-aged adults with obesity assessed the day-to-day variability in insulin areas under the curve, 2-h glucose measurements, and fasting insulin [65]. Subjects were randomly assigned to 1 of 4 exercise regimens: *1*) no-exercise control; *2*) low-amount, low-intensity exercise; *3*) high-amount, low-intensity exercise; and *4*) high-amount, high-intensity exercise [65]. After 24 wk, clinical measure improvement fell within the day-to-day variability across groups, and the authors emphasized the need for more comprehensive measurements of personal activity patterns in dietary interventions [65]. In a longitudinal cohort, increased physical activity was associated with lower BMI and body adiposity index at both baseline and end of follow-up, attenuating the association between *FTO* risk allele rs1421085 and BMI and body adiposity index (see Genetics section for additional discussion on *FTO*). These findings further suggest that regular physical activity can, on the epigenetic level, reduce the burden of genetic predisposition for obesity in certain individuals [66]. In this manner, recording and profiling an individual’s exercise habits can provide a more comprehensive definition of their dietary and interventional needs and can be seen as an important consideration as the field moves toward the design of prospective PN interventional trials. Similar findings were obtained in a large cohort of 18,424 Chinese adults [67], where specific exercises including mountain climbing, walking, exercise walking, dancing, yoga, and jogging were found to attenuate BMI, body fat percentage, waist and hip circumferences [67]. These findings suggest that an individual’s exercise routine, including their intensity, frequency, and personal activity of choice, may be important considerations in the design of PN-based interventions that comprehensively acknowledge the diverse lifestyles of people with overweight and obesity.

## Metabolomics

Metabolomic profiling systematically evaluates many metabolites—endogenous small molecules of cellular biochemistry [68]—resulting in the characterization of various phenotypes as well as new biomarkers of response to diet, important for refining PN-based interventions. Metabolites may arise from the body as well as from human microbiota. Analyzing the content of specific metabolites in the urine and blood can reveal micronutrient deficiencies, provide information about food intake and dietary patterns, and serve to record overall gastrointestinal health [69]. Metabolome analyses reveal the effect and molecular signatures of food and drink processing on the body. For example, proline betaine in the plasma has been identified as a biomarker of orange juice ingestion [70], and 6 biomarkers (ferritin, glycine, diacyl phosphatidylcholines 36:4 and 38:4, lysophosphatidyl choline 17:0, and hydroxy-sphingomyelin 14:1) were associated with meat consumption and diabetes risk, suggesting that unique metabolomic signatures can be used to help identify obesity predisposition [71]. Ultimately, these unique metabolomic parameters can be used to identify dietary patterns. For example, one cohort study of 160 volunteers displayed 3 habitual and distinct dietary patterns as determined through metabolomic analysis of biofluids [72]. In this way, metabolomics provides a highly specific and individualized mechanism or fingerprint of the digestive and metabolic processes that drive fat accumulation and may be integrated into the design of personally tailored dietary interventions.

Additionally, PN-based interventions may utilize metabolomics to assess digestive and metabolic processes to accurately estimate metabolic rate. In humans, predictive equations estimating basal metabolic rate (BMR) have been tested in a variety of cohorts, including high-performance female athletes [73], adult volunteers [74], multiple sclerosis consortium subjects [75], and more. The potential metabolomic identification of BMR may help identify the specific environment and physiological factors that mediate the relationships between an individual’s metabolome and their obesity status. More directly, metabolomic techniques have also been recently validated in identifying a specific metabolite signature for visceral adipose tissue (VAT) deposition [76]. Similar findings have identified increased isoleucine consumption, lower acetate production, and decreased pyruvate/pyroglutamate consumption associated with the VAT metabolic identity, suggesting that glucose metabolic regulation pathways may mechanistically link dietary intake, metabolic processing, and fat deposition [77]. Overall, these techniques provide state-of-the-art information that integrates metabolite fingerprints and individualized metabolic processing pathways with the physiological predisposition to and development of obesity. PN-based dietary interventions may engage these techniques to comprehensively understand an individual’s physiological response to food intake, and in doing so, better adapt interventions to reduce the likelihood of fat deposition and obesity.

There have also been some attempts at utilizing metabolomics to characterize the effects and adherence to specific dietary interventions. For example, the Dietary Approaches to Stop Hypertension (DASH) diet is among the recommended diets for improved cardiovascular health. One study examining serum concentrations of 44 known metabolites found significant variations in the assayed metabolome between patients randomly assigned to a DASH diet and those assigned to a control or fruits and vegetable diet [78]. These serum biomarkers included amino acids (*n* = 1), cofactors (*n* = 2), vitamins (*n* = 2), and lipids (*n* = 41), with N-methylproline, stachydrine, tryptophan betaine, theobromine, and more serving to differentiate the DASH diet [78]. In this case, metabolome profiling helped identify a clear physiological and biochemical phenotype associated with specific dietary intervention and may be utilized to determine adherence to the DASH diet [78]. Similar metabolomic profile variations have been identified in the context of a sodium-restricted DASH diet, which was additionally associated with improved blood pressure and cardiovascular health in 13 patients [79]. Here, metabolite profiles in patients during sodium-restricted dietary intervention suggested that improved energy utilization may mediate the cardiovascular health improvement seen clinically [79]. Taken together, these findings suggest that metabolite profiling may provide a clear biochemical means of assessing adherence and physiological response to specific dietary interventions and serve to optimize the design of interventions based on a person’s individualized likelihood to respond to certain interventions.

Although these findings are promising, the applications of metabolomics to PN remains in its preliminary phases, and large-scale RCTs are needed to determine mechanistic relationships between dietary intake and metabolite profiles, as well as how these metabolite profiles can be used to measure or predict clinical outcomes to certain interventions [80]. Importantly, a lack of evidence in children and adolescents hinders the active integration of these methodologies into PN interventions, and further studies, specifically in pediatric populations, are required to develop early and efficacious interventions against obesity.

## Gut Microbiome

Another key component of gastrointestinal health and disease relevant to the discussion of PN is the gut microbiome, defined as the gut microbiota and its associated genes plus its functional profile [81]. The gut microbiota, of which bacteria have been most studied, are fundamental in modulating the immune response, producing vitamins from diet-derived substrates, digesting nutrients otherwise inaccessible to the host, and maintaining cell membrane integrity [82,83]. With its wide array of physiological functions, the gut microbiome has also been shown to be diverse and highly individualized [84]. Compared to the gut microbiome found in healthy individuals, a “dysbiotic” gut microbiome is often presented as deviations in the relative abundance of specific types of bacteria or reduced diversity; these alterations have also been associated with many disease states including hypertension [85], cancer [86], Alzheimer’s disease [87], and cardiovascular health [88].

Certain gut bacteria may affect the response to dietary interventions in particular individuals [89]. In one study, *Eubacterium xylanophilum*, *Desulfovibrio*, *Terrisporobacter*, *Clostridium sensu stricto*, and *Coprococcus* positively impacted glycemic variability among females with obesity who received a high-protein diet [90]. Here, the authors hypothesized that regulating short-chain fatty acid production mediated an individualized response to high-protein diets [90]. Similarly, the *Dorea* genus of bacteria was also found to be moderately predictive of subsequent weight loss in a trial that, otherwise, found no differences in gut microbiome composition across various dietary interventions [91]. In another study that compared daily energy restriction with intermittent fasting in adults with overweight and obesity, significant intergroup differences in the abundance of *Akkermansia* was found [92]. In this case, these differences and their resulting impact on intervention success were attributed to positive regulation of glucose homeostasis and decreased gut inflammation [92].

Development of gut microbiota-directed foods to target interindividual variation and ultimately improve clinical health outcomes is an active area of research, considering that the gut microbiome appears to have distinct taxonomic profiles in states of either under- or over-nutrition. For example, studies in undernourished children have observed that the gut microbiome is often immature for their chronological age, has lower diversity of microbiota, and/or has higher abundances of inflammation-associated bacteria compared to normal weight children [93,94]; a recent trial examining microbiota-directed complementary foods (MDCFs) in children with moderate acute malnutrition found that children who received complementary foods containing ingredients previously shown to beneficially impact the gut microbiome also had better growth and bone health outcomes [95]. Considering that the gut microbiome has been shown to be altered in individuals with obesity compared to those with normal weight, including lower diversity and richness as well as greater abundance of inflammatory taxa such as Proteobacteria [96], there is potential to design MDCFs that target underrepresented taxa in the gut microbiome during obesity, which ultimately may lead to weight loss. However, more research is needed: randomized trials examining the effect of fecal microbial transfer have failed to show a clinical effect on body weight, and it is unclear whether the taxa associated with obesity are a cause or consequence of excess weight [97].

Overall, the role of the gut microbiome has been linked to the individualized response to dietary interventions [98]. From basic variation in postprandial glycemic responses attributed, in part, to individual differences in microbiome diversity [99] or to the bidirectional relationships between poor diet-microbiome activity-dietary response [100], diverse research suggests a fundamental role of bacterial populations in mediating the individual immune, glycemic, and gastrointestinal response that drives food absorption. Further, a recent randomized trial identified the largest relative variation in gut microbiota following Roux-en-Y gastric bypass against a comparative cohort assigned to medical weight loss or adjustable gastric banding [101]. Specifically, notable increases in *F. prausnitzii* were observed following bypass surgery and medical intervention, whereas microbial diversity decreased following adjustable gastric banding [101]. In this sense, integrating an understanding of the baseline variations in gut microbiome diversity and dysbiosis becomes important in predicting responses to dietary interventions. As such, although the individual variations and baseline differences in microbiome parameters seem indicative of the efficacy of certain diets, further research is needed to better understand the exact role that individualized assessments of microbiome health may play in the development and optimization of precise dietary interventions.

## Behavioral and Socioeconomic Characteristics

Certain behavioral and socioeconomic characteristics are linked to health outcomes including obesity [102]. For example, earning income below the poverty line, being unemployed, and using food stamps was positively associated with obesity prevalence and BMI in one study [103]. Understanding the structural and systemic drivers of overweight and obesity, as well as which behavioral and socioeconomic phenotypes are linked to various obesity-related outcomes, is critical to further refine PN interventions. On the macrolevel, historical and systemic mediators of socioeconomic or political factors—ranging from the neighborhood walkability and district redlining [104] to housing instability and food insecurity [105]—represent key considerations in the equitable delivery of dietary interventions. Unfortunately, given the scope and nature of PN-based interventions, considerations of behavioral and socioeconomic characteristics largely occur at the micro-level, focusing on an individual’s or family’s unique set of circumstances. As such, there has been a recent push to develop machine learning models and psychometric measures that can phenotypically assess psychological and socioeconomic characteristics of the individual in a way in which they may have the potential to inform the development and effectiveness of precision health interventions.

In an attempt to introduce a standardized psychosocial phenotyping methodology, one group applied a novel machine learning approach to determine which psychosocial characteristics were correlated with increased BMI in adults [106]. Here, common phenotypic traits associated with increased BMI included (but were not limited to) insufficient or poor sleep, lack of vigorous activity, and passive engagement with healthcare (limited urge to get second opinions, lack of self-advocacy). Additionally, common phenotypic traits negatively associated with elevated weight included habits of activity, belief in personal responsibility for healthcare, and limited comorbidities [106].

The analysis of psychosocial phenotypes as informative of eating habits and dietary interventions has also been examined in children and adolescents. Here, in toddlers from low-income countries, maternal education, sex, and age predicted eating behavior in absence of hunger (EAH) situations, defined as the continued consumption of foods past satiety [107]. In girls aged 5 to 7, eating behavior in EAH situations remained stable longitudinally, suggesting a lingering psychological inertia involved in dietary behaviors [108]. The psychosocial characteristics of other disruptive eating behaviors including binge eating, caloric restriction, and satiety sensitivity have also revealed the interpersonal variation that predicates a predisposition to obesity [109,110]. Although the categorization of disruptive eating behaviors provides some level of psychosocial nuance, additional implementation of large-scale machine learning models in children and adolescents is needed to create a thorough repertoire of behavioral fingerprints that can associate downstream with having overweight or obesity.

The integration of psychosocial characteristics into the field of precision medicine serves to acknowledge the importance of psychological behavior and socioeconomic factors on the delivery and efficacy of precision dietary interventions but requires much additional study in children and adolescents [111]. Importantly, improved assessment of these considerations may now be feasible with recent technological advances, including the Universal Eating Monitor [112] and Automatic Ingestion Monitor [113], which use standardized methods to assess rates of food consumption, temporal eating patterns, and daily habits. Integrating these novel and standardized assessments of eating patterns and behaviors can provide an additional level of precision for dietary interventions that may then integrate this information to optimize responses to dietary and behavioral interventions. Despite tangible advances in the field’s appreciation of sociological phenomena as mediators of disease, a lack of randomized controlled trials (RCTs) testing these considerations is apparent, and further research is needed to elucidate the optimal integration of individual biogenetic state and sociological characteristics to develop PN interventions. “One-size-fits-all” recommendations and interventions to manage obesity largely fail to consider factors such as income level and socioeconomic status, religion-based food restrictions or preferences, dietary preferences, e.g., veganism, other dietary behaviors, the built environment such as availability of recreational spaces, and access to certain nutritious, high-quality, affordable food—i.e., food deserts. These factors are critical to account for to make any dietary recommendations realistic and actualizable.

## Translating PN: Evidence from RCTs

Key randomized trials examining PN interventions are highlighted here. In 2017, the Food4Me European RCT examined the effect of either standardized virtual dietary advice or virtual personalized dietary advice on dietary intake and anthropometric and blood biomarkers. In this study, personalized dietary advice incorporated insights from baseline dietary information, genetic phenotypes, and baseline anthropometric and blood markers [114]. Results indicated that participants assigned to the precision intervention displayed improvements in dietary behavior (lower consumption of red meat, saturated fat, and salt) and reductions in body weight relative to controls [114]. Although the trial reaffirmed the importance of PN advice, it did not directly assess the efficacy of a directly administered dietary intervention.

In 2021, Ben-Yacov et al. [115] randomly assigned adults with prediabetes to a standardized Mediterranean diet or a personalized postprandial targeting diet (PPT) for 6 mo (*n* = 225) with an additional 6-mo follow-up among a smaller subgroup (*n* = 177). Here, the PPT diet implemented a previously developed machine learning algorithm [19] to use individual clinical and microbiome features to predict the personalized glucose response following a meal [115]. At 6 mo, the trial found significant improvements in individual glycemic control, defined as the time spent with glucose levels >140 mg/dL or 7.8 mmol/L, for participants administered the PPT diet relative to the standard Mediterranean diet, which was maintained at the 12-mo follow-up [115]. Average body weight decreased by 2.9% and 3.5% of baseline weight in the Mediterranean diet and PPT diet, respectively, but there was no significant difference between groups at 6 or 12 mo. Adverse events were not related specifically to the personalized nature of the interventions and included tolerance and side effects to either diet (bloating, indigestion) or in a small group of participants, allergy to the adhesive from continuous glucose monitoring sensors connected to each participant. This study provides evidence of the utility of PN interventions in addressing the glycemic dysregulation characteristic of diabetes among individuals with prediabetes and further affirmed the need to better understand PN interventions against “one-size-fits-all” dietary interventions.

A similar 6-mo randomized trial to investigate the effect of precision dietary interventions compared to the one-size-fits all approach was recently published [116]. In this study, 204 adult participants were randomly assigned to either a low-fat standardized diet (<25% of caloric intake) or a personalized diet predicting postprandial glycemic response with machine learning algorithms [116]. Here, both groups displayed negative changes in weight across the 6 mo of intervention (control: −4.31%, personalized: −3.26%), with no differences in weight loss between groups [116]. This trial’s results indicated no variation between a personalized diet and standardized intervention, possibly due to low adherence to the study interventions. Adverse events were not reported. These mixed findings reinforce the need for additional RCTs with large and diverse samples and longer follow-up periods to consolidate the literature and to include children and adolescents with obesity.

Comparisons of nutrition-based precision interventions with baseline placebo supplements have also been conducted through the Personalized Prevention of Colorectal Cancer Trial (PPCCT), in which the magnesium status of 250 participants at high risk of colorectal cancer was related to vitamin D status [117], medium-chain fatty acids (MCFAs), and the gut microbiome [118]. In 2018, researchers assigned the PPCCT cohort to either a customized magnesium supplementation optimized to baseline dietary intakes or a placebo group and measured the effect on baseline 25-hydroxyvitamin D [25(OH)D], a product and regulator of vitamin D catabolism [117]. Here, baseline 25(OH)D significantly impacted the effect of magnesium supplementation, with increases in 25(OH)D postsupplementation, when baseline concentrations were ∼30 ng/mL and decreases in 25(OH)D postsupplementation with baseline concentrations >30 ng/mL [117]. These findings place great importance on the individual variation of metabolites in mediating the impacts of supplementation and provide evidence for a need to integrate these personalized dietary factors in supplementation trials. In a recent extension of the PPCCT, Fan et al. [118] examined the effect of personalized magnesium supplementation and a placebo control on blood glucose, plasma MCFAs, citric acid cycle metabolites, and microbiome status in fecal samples. Findings indicated that personalized optimization of the Ca:Mg ratio increased circulating levels of MCFAs, resulting in important biochemical downstream effects such as improved microbiota-mediated metabolism. Overall, these studies highlight the important integration of a precision-based framework into the effective design of nutrition interventions, with individual variations of circulating metabolites, physiological risk, or mineral baselines serving as important factors contributing to intervention response and efficacy. Although the PPCCT marks a recent and ongoing effort to implement PN interventions in clinically relevant groups, further research must expand into children and adolescent groups and provide an added emphasis of biochemical and mechanistic links.

Most recently, the relationship between tissue-specific insulin resistance and individual dietary modulation was studied in a 12-wk PN trial. Here, 242 adults were categorized into groups based on muscle-insulin resistance (MIR) and liver insulin resistance (LIR) [119]. Participants then followed either a high-monounsaturated fatty acid diet (HMUFA) or a low-fat, high-protein, and high-fiber diet (LFHP). Individuals with MIR and a HMUFA diet alongside participants with LIR and LFHP diets were considered to be part of the phenotypic diet group A (PhenoDiet A) [119]. Individuals with MIR and an LFHP diet alongside subjects with LIR and a HMUFA diet were considered to be part of the phenotypic diet group B (PhenoDiet B) [119]. With this methodology, tissue-specific insulin resistance phenotype was used to assess differential response to dietary intervention. The trial found clinically significant improvements in insulin sensitivity, triglyceride concentrations, fasting plasma insulin, glucose tolerance, and C-reactive protein in PhenoDiet B relative to PhenoDiet A, suggesting that integrating insulin resistance phenotype into dietary intervention design can concretely improve cardiometabolic health [119]. Overall, the trial provided a proof-of-concept that insulin resistance phenotyping can be used to induce clinically pronounced improvements in metabolic health, reiterating the efficacy of PN-based dietary interventions. Future trials may expand the physiological breadth of individualized phenotyping, further optimizing the clinical improvements mediated by PN.

## On the Horizon: Ongoing Investigations of PN

**FIGURE 2:**
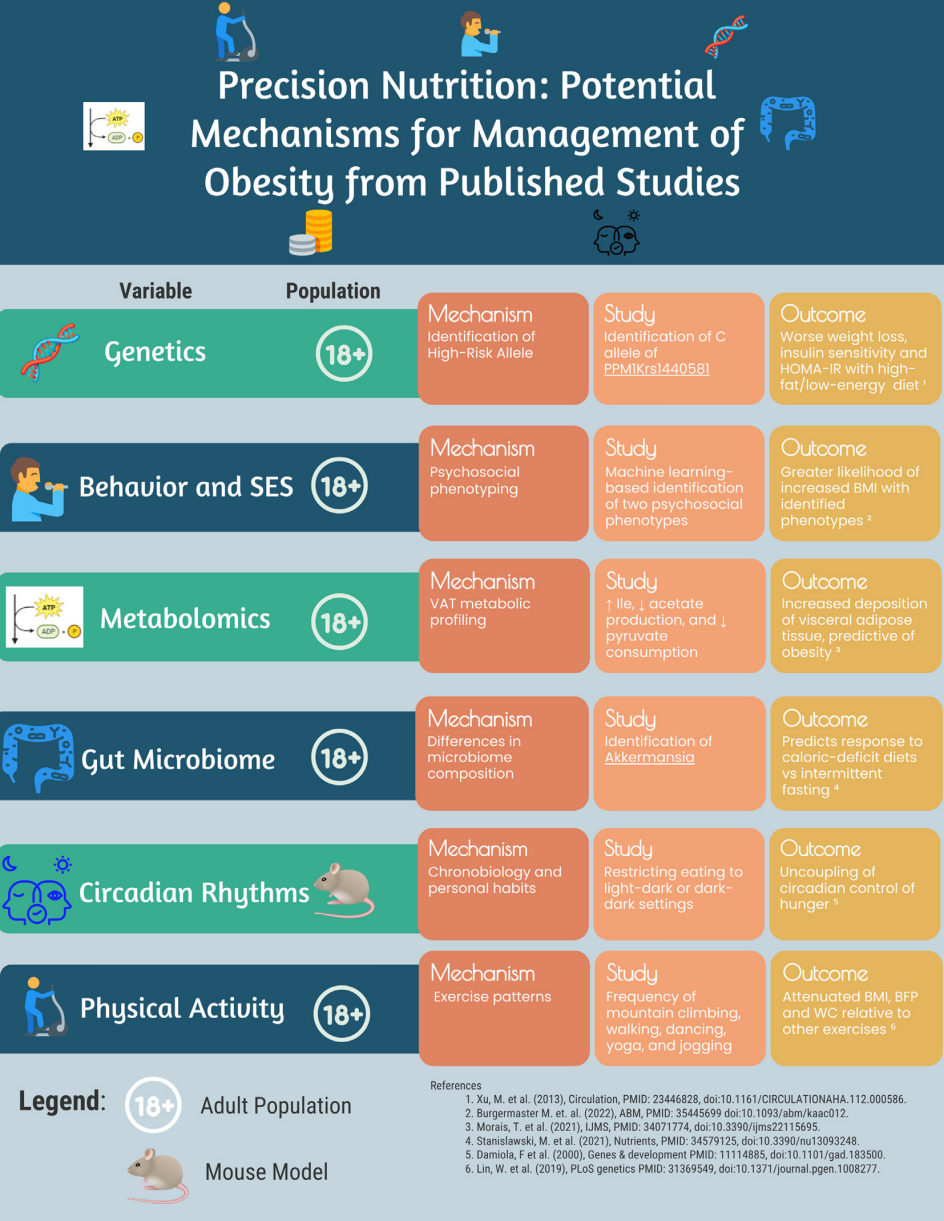
Potential mechanisms for PN interventions. Sample mechanisms across 6 domains of consideration, including genetics, behavior/socioeconomic status, metabolomics, gut microbiota, circadian rhythms, and physical activity are displayed. All studies using human subjects were conducted in high income countries. BFP, body fat percentage; Ile, isoleucine; PN, precision nutrition; SES, Socioeconomic status; WC, waist circumference.

Importantly, there are many ongoing clinical investigations of personalized dietary interventions and assessments that are aimed at clarifying the role of these domains on anthropometric and other health-related outcomes. For example, one Romanian based trial is currently underway to determine how the relationships between genotype and nutrient plasma levels can be normalized via PN intervention (NCT05342766). Further attempts at assessing PN-based interventions include implementation of machine learning algorithms, with one ongoing trial using these technologies to use dietary intake, nutritional status, and other markers to tailor dietary interventions for maximal efficacy (NCT05701657). Similarly, another ongoing trial is aimed at determining optimal dietary strategies for participants with variations in metabolic phenotype, focusing on tailoring interventions to metabolic function and cardiovascular health (NCT04131166). Finally, there are multiple ongoing investigations assessing PN-based dietary interventions for therapeutic benefit in metabolic diseases. These include the PRECISION_T2D study, which will attempt to integrate gut microbiota profiles and glucose metabolic profiles to assess responses to specific dietary interventions (NCT05885828), as well as the VIOME Precision Nutrition Program, which will use personalized diets based on questionnaires, blood, stool, and saliva-based assays to reduce HbA1c (NCT06185192). Although these trials represent significant investment and advancement of the field, there persists a great need for additional RCTs to assess the efficacy of PN, with a focus on integrating various clinical, demographic, and biophysiological domains. Overall, as the current literature on PN continues to identify potential targets and mechanisms (Figure 2), future research must address personal glycemic response, microbiome diversity, genetic predisposition, socioeconomic factors, circadian rhythms, and psychosocial characteristics in precision dietary interventions—and in addition, must involve longer follow-up periods to determine long-term sustainability of effect. Although RCTs have started to implement nutrition-based precision medicine interventions in relatively large cohorts, further research is required to integrate clinical insights with population-based interventions. Such efforts are being carried out by, for example, the recent Nutrition for Precision Health initiative funded by the NIH, which aims to examine how individuals respond to different diets and whether this response can be predicted using novel artificial intelligence and machine learning methods.

## Limitations

Despite large-scale efforts to advance the optimization and implementation of PN interventions, numerous limitations must also be acknowledged. These may be binned as *1*) more data or evidence needed for a given domain, such as circadian rhythms; *2*) better methods for harmonizing complex multimodal data needed as well as their analysis and interpretation, such as genetics; and *3*) better translation into guidelines and implementation, such as trials examining multiple-domain-predicted interindividual glucose responses, across diverse populations.

Although our review emphasizes the need to consider the aforementioned domains in the design and implementation of PN interventions, there is considerable variation in the methodology, design, and strength of evidence across the 6 domains. For example, the genetic predisposition to obesity and its potential utility in optimizing tailored interventions has been widely documented in animal models and human studies. However, examination of circadian rhythms and temporal variations in dietary patterns has been largely limited to in vitro analyses and animal studies. Other domains, such as physical activity, metabolomics, and the gut microbiota, are supported largely in theory by correlative studies connecting clinical outcomes to dietary patterns. As such, primary randomized clinical and nutritional interventions need to be conducted to truly determine whether the consideration of these 6 domains is warranted in practice.

In the large-scale implementation of PN, logistical questions of informed consent, data management, and the process of optimization seem ample [120,121]. Although PN interventions attempt to target the individual variations in obesity diagnosis and progression, they largely fail to address the large-scale systemic factors that impact public health: healthcare systems, systemic racial inequity, and population food distributions and supply chains. As such, tailored interventions at the individual level may fail to address global obesity prevalence if it cannot address the cultural, economic, and political variations of these systemic factors. Similarly, it is unclear whether PN can be effectively utilized in low- and middle-income countries, where healthcare infrastructure and the financial feasibility of these extensive interventions become questionable and questions of equitable access to PN interventions become apparent. The ongoing development of PN-based intervention also raises a variety of important ethical concerns, including concerns of family dynamics, access to resources, and cultural beliefs toward scientific tests of genetics and metabolomics [122]. Further work is needed to ensure that PN interventions are deliverable to all socioeconomic and cultural populations [123]. The optimization and design of PN interventions also raises the important question of technical feasibility and implementation. As previously mentioned, the strength of evidence and readiness for cooperative implementation across variables into PN interventions is highly varied across the 6 domains, and current technologies are likely not sensitive, standard, and powerful enough to optimally integrate all 6 domains. Although machine learning models and the ongoing development of generative artificial intelligence may provide the computing power and technological precision needed to integrate individualized factors in the design of a dietary intervention, there are sustained barriers to implementation including incomplete standardization and characterizations based on these domains, sample acquisition, and personalized dataset construction. Given these considerations, it becomes clear that the substance of these 6 domains of PN must be examined alongside the evolving technological landscape of precision interventions to truly push the field forward.

Additionally, as evident from all 6 domains of PN, current evidence and research on PN and obesity prevention largely centers on adult populations. Given the novelty of the field and a dearth of studies on children and adolescents, further work is needed to address how PN dietary interventions can be used to specifically target obesity and its related biophysiological complications in children. Future prospective and retrospective studies in children and adolescents may attempt to integrate one or more of these 6 key domains and provide an early and unique perspective on our ability to control overweight and obesity prevalence in pediatric populations. In short, the future of PN must consider the physiological and socioeconomic factors that influence obesity at the individual level while also acknowledging the need for whole-scale reevaluations of global health disparities, nutritional outcomes, and healthcare access.

## Future Directions and Conclusions

Over the last decade, there has been an increased and warranted focus on addressing obesity using PN and personalized dietary interventions. In considering the pathogenesis and prevalence of obesity, dietary interventions must continue to acknowledge the importance of genetic variation, social conditions, biological circadian rhythms, and psychosocial phenotypes in mediating obesity. Optimizing standardized dietary interventions at the individual level through genetic and microbiome testing, lifestyle pattern analysis, and phenotyping may help advance our ability to optimize diets to the highly nuanced and individualized physiological factors involved in obesity, and in doing so, inform the development of state-of-the-art PN methods. PN research requires tools that enable accurate nutritional impact prediction on health using -omics technology, enhanced patient bioinformatics and database management, precise biomarkers, microbiome and metabolomics assessment of disease progression, and patient response to nutritional treatment [124]. Although we categorize relevant information across 6 domains, this is largely done to facilitate organization of current considerations for PN-based interventions, and there is certainly overlap across these domains. Over the coming years, we anticipate rigorous evidence on other macro-influences, such as climate change, food system architecture, and global geopolitics, to ultimately add to the puzzle of PN. As a result, integrating the 6 domains in the development of PN interventions is crucial in providing a comprehensive and effective approach against obesity. Considering assessments of the gut microbiota, metabolomics profiling, and genetics enables personalized dietary recommendations that incorporate individualized physiological parameters that may contribute to obesity pathogenesis, response to interventions, and clinical outcomes. Further, inclusion of socioeconomic criteria that can influence dietary choices, eating habits, and access to resources allows for further tailoring of interventions to an individual’s socialized nutritional landscape. Finally, integrating characterizations of an individual’s physical activity patterns and circadian rhythms may help optimize the temporal delivery and management of dietary interventions, acknowledging the role of daily habits and variations in mediating the development of and response to obesity. The future of PN interventions may lie in the holistic integration of these 6 domains, providing a multifaceted approach that combines habits and daily variations with external pressures and internal physiology to develop a comprehensive approach to obesity management. As artificial intelligence and machine learning methods evolve to be able to incorporate and analyze complex multimodal data, our models for PN will improve.

With these considerations in mind, future research must involve additional RCTs that compare precision dietary interventions to one-size-fits-all dietary interventions with long-term follow-up, effectiveness studies to assess the viability of these interventions in real-world settings, as well as methodologic studies characterizing the nuanced development of PN. In children (Box 1), challenges in obtaining appropriate biological samples warrant further research to better understand the -omics analyses of less invasive biological sampling including urine, saliva, and hair. Particularly, socioeconomic and physiological predispositions to obesity and other metabolic conditions place children at further risk, and an emphasis on understanding the pathogenesis of these conditions in children as well as the effects of dietary interventions in this high-risk population is needed. Currently, there is sparse evidence of PN and other dietary interventions in these groups, and careful consideration of nutritional physiology and intervention to prevent overweight and obesity in high-risk subgroups is needed. As such, there is a need to study and implement integrated nutrition-based precision medicine interventions in children, adolescents, and other groups at risk of poor nutrition, and future research should attempt to deliver these interventions to a wider array of populations. Clarifying the role of these interventions in high-risk populations may provide an early and efficacious means of combating obesity at its source, providing clinical benefits across the age spectrum by targeting metabolic diseases in childhood that are often the source of numerous complications that increase morbidity, mortality, and reduce the quality of life.

BOX 1 **Recommendations for obesity management in children**

• In 2023, the American Academy of Pediatrics released the first edition of clinical practice guidelines for evaluation and management of children and adolescents with obesity [125].
• These guidelines recommend that behavioral and lifestyle changes should be the first-line approach to combat obesity in children [126].
• The guidelines also note that there is no evidence to support “watchful waiting” and the unnecessary delay of treatment; instead, weight loss pharmacotherapy should serve as an adjunct to health behavior and lifestyle treatment in adolescents as young as 12 y old with BMIs ≥95^th^ percentile [126].
• Further, the guidelines indicate that the existing data show metabolic bariatric surgery is safe for adolescents older than 13 y with severe obesity (BMI ≥120% of the 95^th^ percentile for age and sex) [126].
• The WHO plans to release guidelines on the prevention and treatment of obesity in children and adolescents, specifically in the 0 to 9 and 10 to 19-y-old age groups, for its Member States in the near future [127].
• NIH’s Strategic Plan for Nutrition Research for 2020–2030 emphasizes the role of PN, and though the evidence to date is limited, there are multiple studies published or underway, mostly in adults but some in children [21].
• The limited evidence thus far along with its mechanistic basis in summarized in this article (see “Translating PN: Evidence from RCTs”); a number of studies are currently underway particularly in children [[128], [129], [130]].

Alt-text: BOX 1

## Author contributions

The authors’ responsibilities were as follows – SM: was primarily responsible for the design, writing, and final content of the manuscript; NHM, SLH, SM: wrote the first draft; RK, JPPR, JLF, SK: reviewed and edited the draft; and all authors: read and approved the manuscript.

## Funding

The Department of Nutrition and Food Safety at the World Health Organization (WHO) commissioned and provided financial support for this work. The Department of Nutrition and Food Safety at WHO acknowledges financial support from the Norwegian Agency for Development Cooperation (NORAD), the Swedish International Development Cooperation Agency (SIDA), the Government of the Grand Duchy of Luxembourg, and the Government of Germany (BMG). SLH is supported by the NIH under award 5T32HD087137.

### Conflict of interest

SM has an equity interest in VitaScan, a startup seeking to commercialize technology for point-of-care assessment of micronutrient status partially based on his research as a faculty member at Cornell University. The content is solely the responsibility of the authors and does not necessarily represent the official views of the Eunice Kennedy Shriver National Institute of Child Health and Human Development (NICHD) or the National Institutes of Health. Juan Pablo Peña-Rosas is a full-time staff member at the World Health Organization. The review authors alone are responsible for the views expressed in this publication, which do not necessarily represent the official position, decisions, policy, or views of the WHO. All other authors report no known conflicts of interest.
